# Pediatric free fillet of leg flap for external hemipelvectomy coverage: A case report and systematic review

**DOI:** 10.1016/j.jpra.2025.12.032

**Published:** 2026-01-07

**Authors:** Joshua Khorsandi, Michael Kahen, Justin Kahen, Evan J. Childers, Matthew Kelecy, John P. Brosious, John deVries, Joshua J. Goldman

**Affiliations:** a Kirk Kerkorian School of Medicine at University of Nevada Las Vegas, Las Vegas, NV 89106, USA; b Department of Plastic and Reconstructive Surgery, University of Nevada Las Vegas, Las Vegas, NV 89106, USA; c Department of Surgery, University of Nevada Las Vegas, Las Vegas, NV 89106, USA; d Department of Orthopedics, University of Nevada Las Vegas, Las Vegas, NV 89106, USA

**Keywords:** External hemipelvectomy, Free fillet-of-leg flap, Pediatric pelvic reconstruction, Microvascular free tissue transfer, Spare-parts reconstruction

## Abstract

**Background:**

External hemipelvectomy leaves large composite defects that are difficult to reconstruct. The free fillet of leg flap repurposes soft tissue from the amputated limb to provide durable coverage without additional donor-site morbidity. Pediatric applications are rarely reported.

**Methods:**

We conducted a PRISMA-guided systematic review (1999–2025) across PubMed and Google Scholar. Inclusion criteria were external hemipelvectomy reconstructed with a free flap and reporting at least flap survival and/or complications. Data were extracted at the case and study levels. We computed pooled proportions (Wilson 95% Confidence Intervals) for primary outcomes (flap survival/complications) overall and by flap category. We complement this synthesis with a detailed pediatric case from our institution.

**Results:**

Forty-three studies comprising 94 cases met inclusion. Among cases with explicit reporting, pooled flap survival was 95% (65/69; 95% CI 86.0–97.7), and any complication occurred in 44% (28/64; 95% CI 32.3–55.9). By flap category, survival was reported in 36/38 fillet-of-leg, 7/8 fillet-of-leg with fibula only, 3/3 fillet-of-leg with fibula and tibia, 7/7 anterolateral thigh (ALT)/anterior thigh, 6/6 latissimus dorsi, 2/3 medial thigh, 3/3 other/unspecified, and 1/1 free vascularized fibular graft (FVFG) cases. Complications occurred in 14/34 fillet-of-leg, 5/7 fillet-of-leg with fibula only, 2/3 fillet-of-leg with fibula and tibia, 1/7 ALT/anterior thigh, 3/6 latissimus dorsi, 1/3 medial thigh, 3/3 other/unspecified, and 1/1 FVFG reconstructions. Our case involved an 11-year-old boy who underwent external hemipelvectomy with a free fillet-of-leg flap, achieving full healing by 6 weeks and ambulation by 5 months.

**Conclusions:**

Fillet-of-leg free flaps offer reliable, donor-site-sparing coverage for hemipelvectomy defects with high flap survival. This pediatric case supports feasibility and highlights strategies to minimize ischemia and manage venous congestion.

## Introduction

External hemipelvectomy—en bloc resection of the hemipelvis with the ipsilateral lower extremity—remains a limb-sacrificing but indispensable operation for locally advanced pelvic malignancy and select non-oncologic indications.^1^ The resulting composite defect exposes intra-abdominal and retroperitoneal structures and demands reconstruction with large volumes of durable, well-vascularized tissue to permit early sitting, transfers, and eventual prosthetic rehabilitation while minimizing added morbidity.^2^ Contemporary series emphasize that reconstruction after hemipelvectomy is technically demanding, with wound complications a dominant risk and outcomes closely tied to flap selection and perioperative planning.^3^

Over the past two decades, lower-extremity free fillet of leg flaps (FFLFs)—which repurpose viable tissue from the amputated limb—have gained prominence because they provide substantial bulk without creating a new donor site, conform well to pelvic contours, and can facilitate rehabilitation.^4^ Cohort and case-series literature specifically addressing hemipelvectomy reports reliable soft-tissue coverage and functional recovery with free fillet flaps used either alone or within broader reconstructive algorithms, supporting their role as a mainstay option when local pedicled tissue is insufficient.^4^

Institutional experiences spanning many years underscore the feasibility of lower-extremity fillet flaps (free and pedicled variants) for complex hemipelvectomy defects, documenting durable coverage and acceptable complication profiles in heterogeneous oncologic populations.^5^ These data also highlight that most evidence derives from case reports and small series, reinforcing the need for transparent reporting of endpoints when counseling patients and planning multidisciplinary care.^6^

A central technical priority in FFLF reconstruction is minimizing ischemia time through meticulous coordination of simultaneous flap harvest and tumor extirpation.^7^ Streamlined approaches tailored to hemipelvectomy have been described to shorten ischemia intervals and improve flap reliability—an especially relevant consideration in long, hemodynamically variable oncologic procedures.^8^

Historically, the conceptual basis for using tissue from an amputated limb to reconstruct massive pelvic defects dates to early reports of free fillet lower-leg flaps for pelvic reconstruction, which established the practicality of this donor-site-sparing strategy and paved the way for subsequent refinements.^4^ Building on that foundation, contemporary series applying fillet flaps specifically after hemipelvectomy have validated their capacity to deliver robust, well-vascularized coverage with low donor-site burden in appropriately selected patients.^2^

Against this background, we report an 11-year-old patient who underwent external hemipelvectomy reconstructed with an FFLF and present a PRISMA-guided systematic review of free-flap reconstruction after hemipelvectomy. By pairing a detailed case with pooled outcome estimates across flap categories and explicit accounting of reporting completeness, we aim to provide pragmatic evidence to support decision-making and perioperative strategy selection in this rare but consequential reconstructive setting.

## Case report

### Patient and preoperative course

An 11-year-old boy with a prior excision of inflammatory myositis of the left thigh developed a rapid symptomatic recurrence within 6 months. Core biopsy confirmed inflammatory myofibroblastic tumor with poor response to systemic chemotherapy. After multidisciplinary tumor board discussion, external hemipelvectomy was recommended for local control. Plastic surgery was engaged preoperatively to plan immediate soft-tissue coverage with a free fillet of leg flap (FFLF). The family was counseled about limb sacrifice, reconstructive options, expected rehabilitation, and risks including flap failure and wound complications.

### Operative strategy and technique

**Figure 1:**
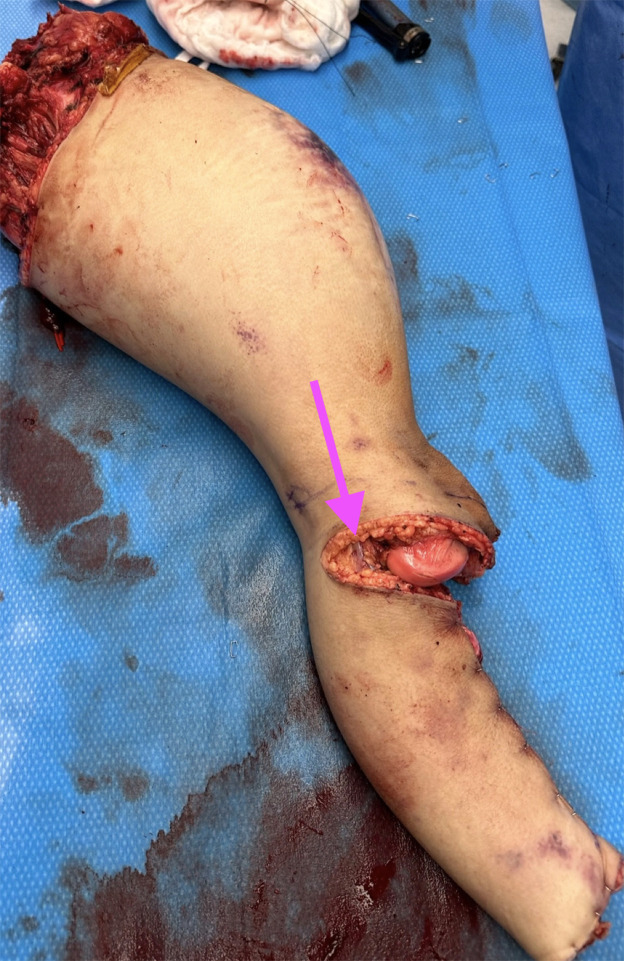
Amputation specimen demonstrating the harvested free fillet of leg flap following external hemipelvectomy, showing intact soft-tissue envelope and preserved popliteal vessels for microvascular transfer. The arrow indicates the popliteal vessels exposed within the amputation stump.

**Figure 2:**
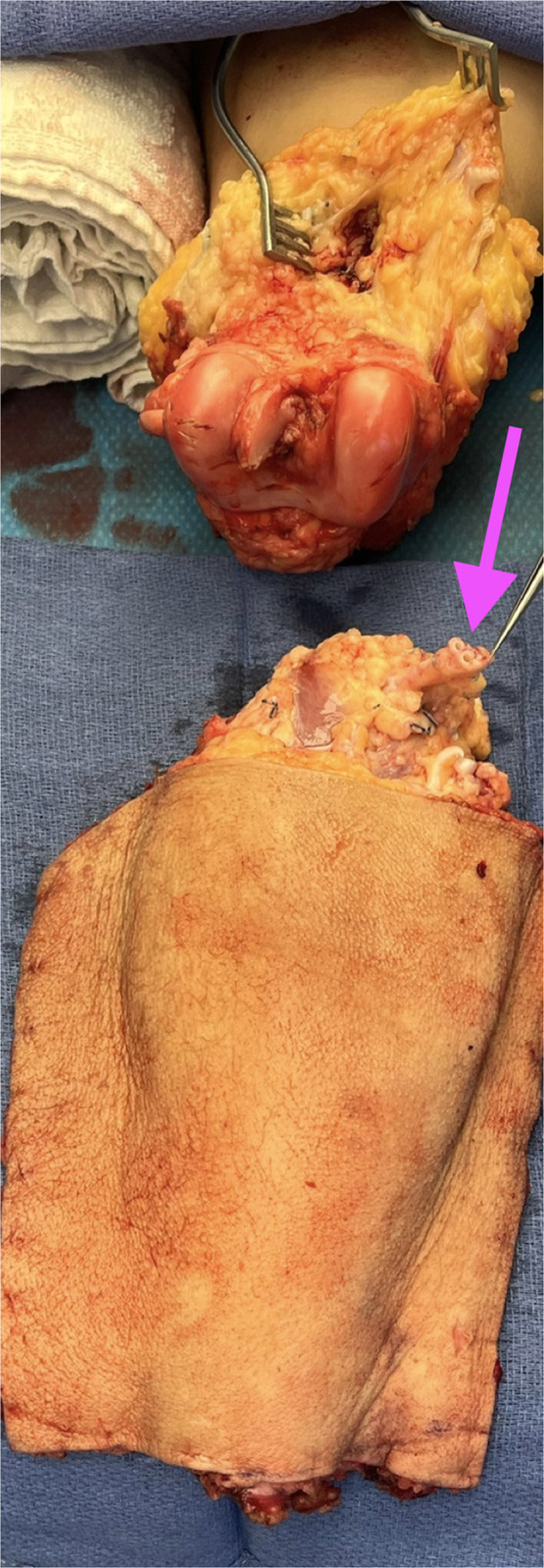
Harvested free fillet of leg flap showing preserved popliteal artery and vein pedicle prepared for microvascular anastomosis following tumor extirpation. The arrow highlights the popliteal vessels following further dissection.

Under general anesthesia with arterial/central monitoring, the patient was positioned supine to allow a true two-team approach. While orthopedic and surgical oncology teams performed the extirpation, (Figure 1) the plastic surgery team prepared the FFLF to minimize global ischemia time. Skin markings encompassed tissue from the distal popliteal crease to approximately 5 cm proximal to the ankle to optimize bulk while preserving reliable vascular anatomy. After skeletal resection, the distal leg vessels were divided and the foot amputated (Figure 2). The popliteal artery and vein were dissected circumferentially and carried proximally to the tibioperoneal trunk to maximize pedicle length and caliber. The flap was wrapped in moist towels on a back table while recipient vessels were prepared.

**Figure 3:**
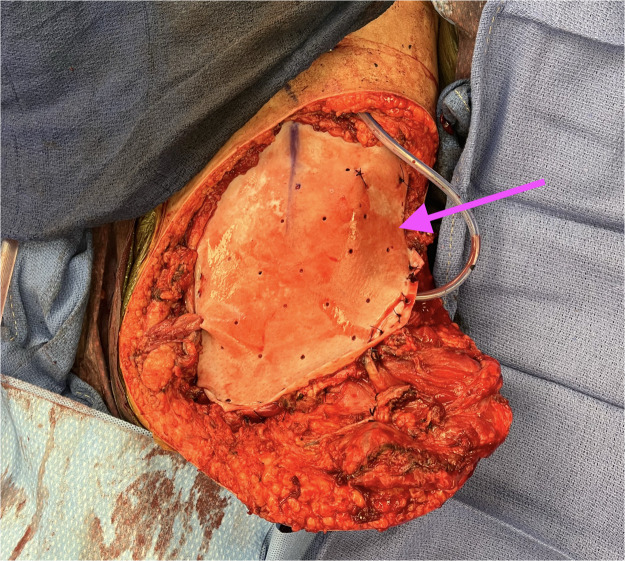
Intraoperative view showing inset of the free fillet of leg flap into the hemipelvectomy defect with biologic mesh reinforcement and drainage in place, providing durable soft-tissue coverage over exposed pelvic structures. The arrow denotes the biologic mesh secured over the reconstructed defect.

The resultant hemipelvectomy defect measured 25 × 19 cm with exposed abdominal contents and spermatic cord. A tailored biologic mesh was inset to protect the viscera, with fenestrations fashioned for the spermatic cord and the pedicle to prevent compression (Figure 3). The popliteal artery was anastomosed end-to-end to the external iliac artery, and the popliteal vein to the common iliac vein, providing a straight line to facilitate venous egress. After warm saline reperfusion and hemostasis, the flap demonstrated appropriate capillary refill and strong audible Doppler signals. Contouring was performed to obliterate dead space and recreate a stable sitting surface. Closed-suction drains were placed, and the inset was completed in layered fashion with meticulous attention to tension-free closure and pedicle protection.

### Early postoperative course

**Figure 4:**
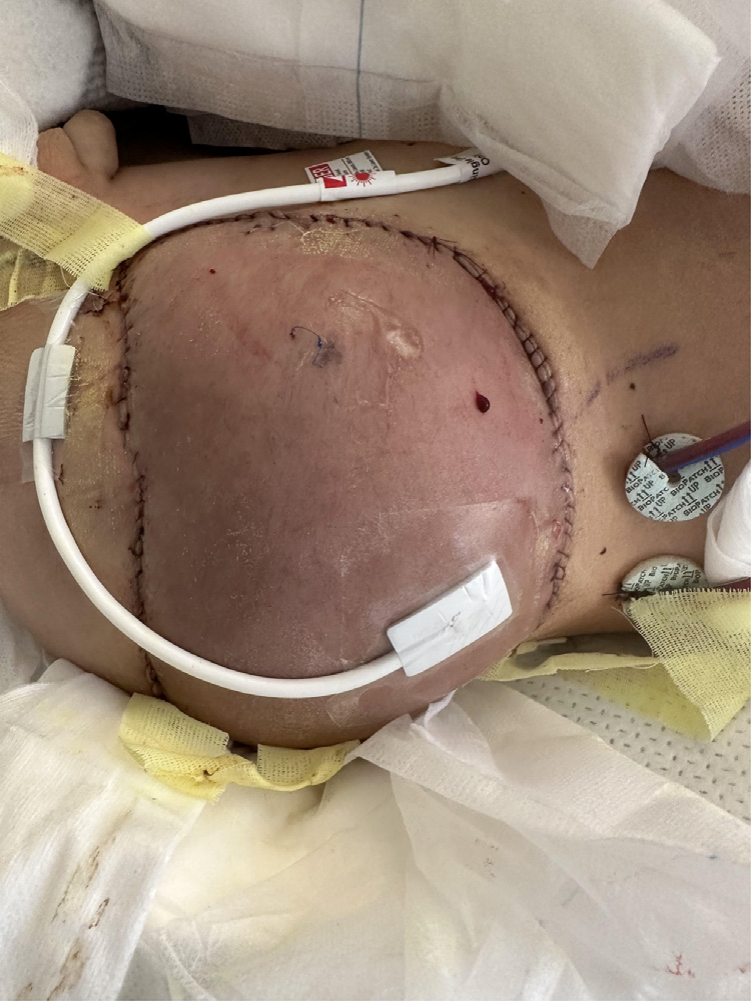
On postoperative day 2, the flap appears tense and mildly violaceous with intact circumferential sutures. Patchy duskiness and mottled discoloration across the surface are consistent with early venous congestion.

**Figure 5:**
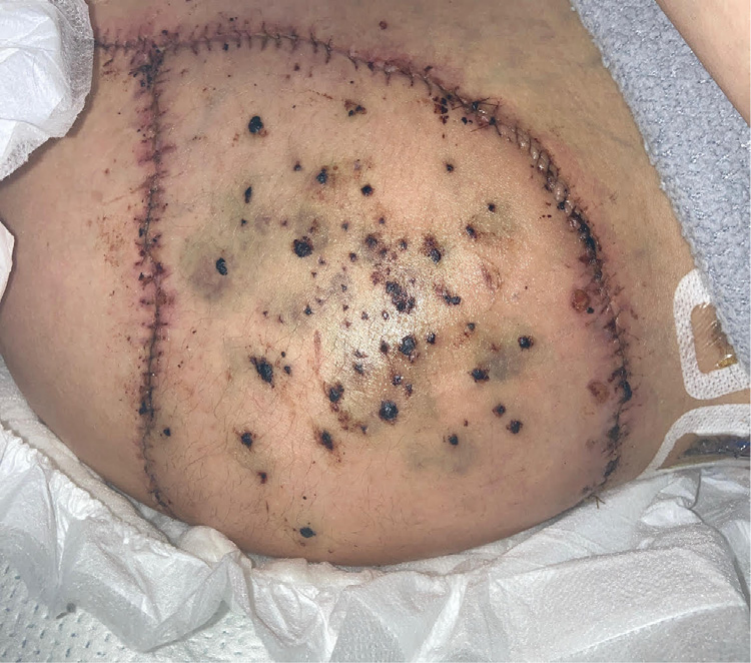
At roughly 2 weeks postoperatively, just prior to discharge, the flap shows a stable contour and uniform pink coloration with fading ecchymoses. Small hyperpigmented marks from prior leech sites are still visible, and the incision lines are fully healed, indicating successful flap recovery.

The patient was monitored in a step-down setting with frequent flap checks (clinical exam and handheld Doppler). On postoperative day (POD) 2, progressive violaceous discoloration and turgor suggested venous congestion (Figure 4). Return to the operating room on POD 3 revealed patent arterial and venous anastomoses with no mechanical kinking or thrombus. Given the distended abdomen and reduced bowel sounds, congestion was attributed to elevated intra-abdominal pressure from postoperative ileus impeding venous return. A stricter bowel regimen was instituted, and medicinal leech therapy was applied for 5 days with close laboratory surveillance and transfusion thresholds individualized to maintain safe hemoglobin levels. The congestion gradually resolved (Figure 5). No wound dehiscence or infection occurred, and drains were removed as output declined.

### Rehabilitation and follow-up

**Figure 6:**
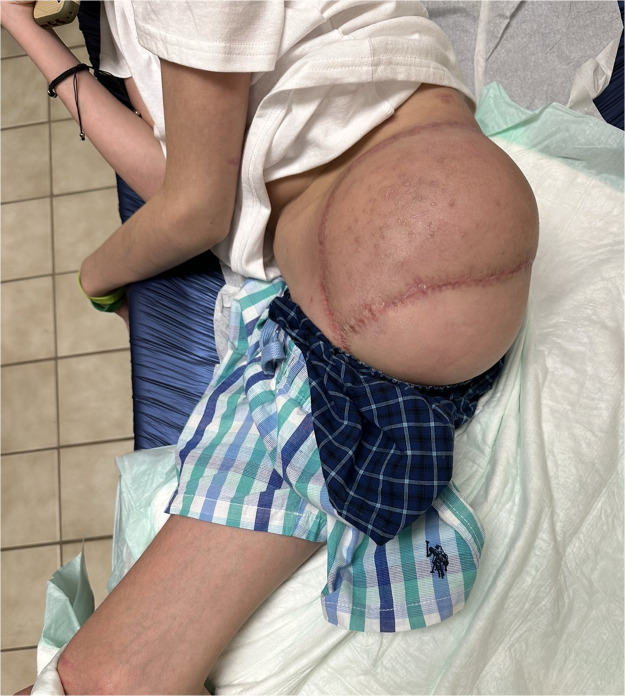
Six-week postoperative appearance demonstrating complete healing of the free fillet of leg flap (FFLF) with well-contoured soft tissue and stable coverage, allowing comfortable sitting and early rehabilitation.

**Figure 7:**
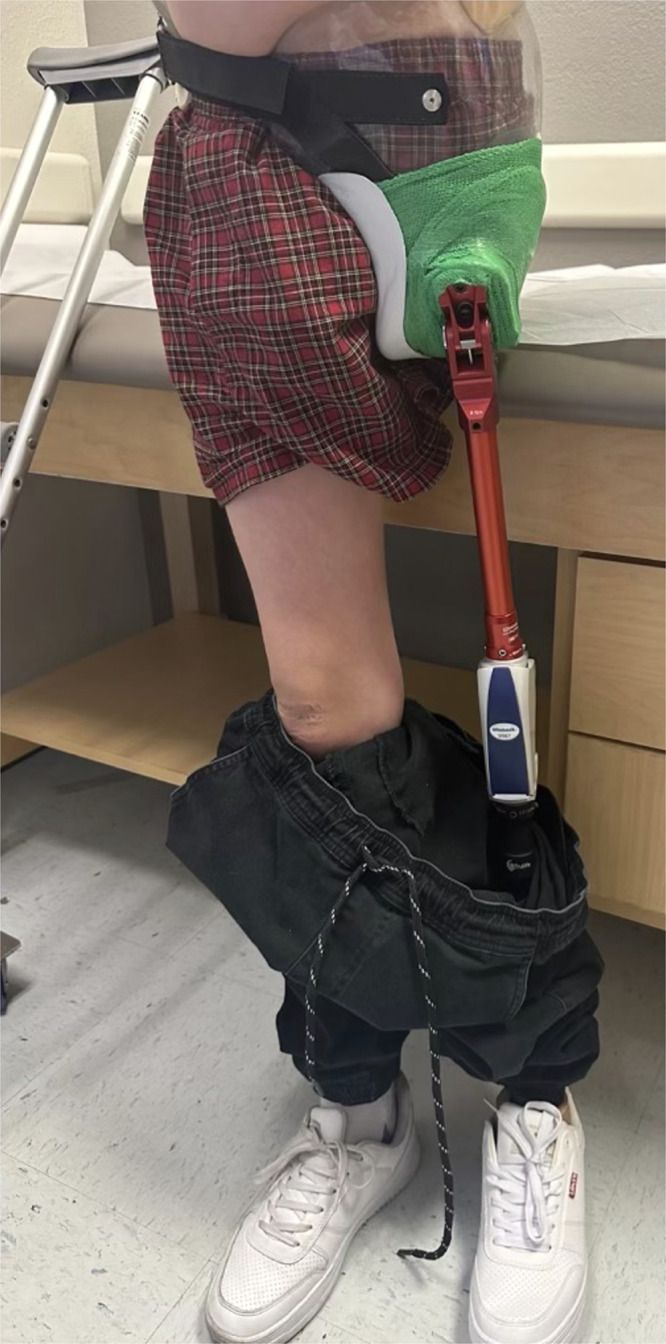
Five-month postoperative image showing successful prosthetic fitting and ambulation following external hemipelvectomy reconstructed with a free fillet of leg flap.

Bed mobility and sitting tolerance progressed during week 1, with protected off-loading of the flap and gradual increase in upright time. At 6 weeks, the incision was fully epithelialized and the flap was soft and pliable, allowing initiation of gentle core and contralateral limb strengthening (Figure 6). At 3 months, the flap was completely healed and volume stable; residual contouring with compression garments improved socket interface planning. At 5 months, the patient ambulated with a custom prosthesis and a single crutch (Figure 7), performing household and short community distances with supervised physical therapy. The patient and family reported satisfaction with comfort while sitting, hygiene access, and early return to school activities.

## Methods

### Protocol and reporting

We conducted a prespecified, PRISMA-2020–conformant systematic review (protocol internal, not externally registered). Eligible studies described external hemipelvectomy reconstructed with free flaps, including fillet variants. Our primary outcomes were flap survival and any postoperative complication; secondary endpoints included functional outcomes, length of stay, and mortality. Comparisons across flap types were summarized descriptively rather than through formal comparative meta-analysis.

### Information sources and search strategy

We systematically searched PubMed and Google Scholar from database inception through September 20, 2025. A representative PubMed string was: (“fillet flap” OR “free fillet flap” OR “fillet of leg” OR “fillet lower leg” OR “fillet of thigh”) AND (“hemipelvectomy” OR “external hemipelvectomy” OR “hip disarticulation” OR “pelvic reconstruction”). Google Scholar was queried with analogous terms. The combined search identified 273 titled records. Cross-database overlaps yielded 19 duplicates at identification, with an additional 7 within-set duplicates removed during screening for a total of 26 duplicates. This left 247 unique records for screening, of which 43 studies (94 cases) met inclusion criteria, and 230 were excluded for prespecified reasons (e.g., no hemipelvectomy mentioned, no primary cases, pedicled-only reconstructions).

### Eligibility criteria

**Figure 8:**
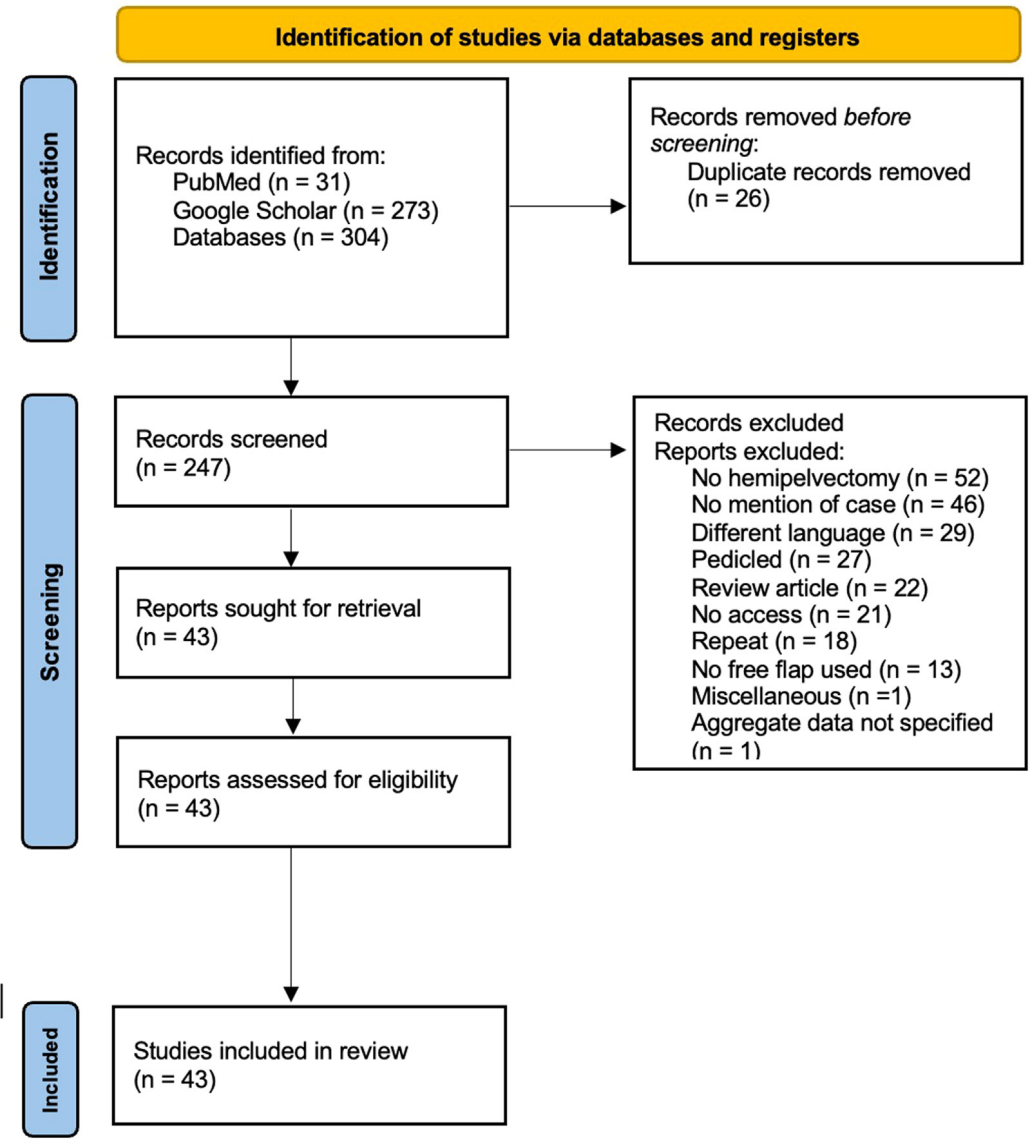
PRISMA flow diagram summarizing study identification, screening, exclusion, and inclusion for the systematic review (*n* = 43 included studies).

Studies were included if they described patients undergoing an external hemipelvectomy or an equivalent high-level pelvic amputation that was reconstructed with a free flap for soft-tissue coverage. We excluded studies limited to pedicled flaps, amputations outside the pelvis, review articles without primary cases, and reports that lacked sufficient outcome data (Figure 8).

### Study selection and data collection

Titles and abstracts were screened, with full texts reviewed when available. We abstracted study‑ and case‑level characteristics: year, setting, cases per study, age, sex, side, indication, flap category (free fillet of ALT/AT, free fillet of latissimus dorsi, free fillet of lower leg (unspecified), free fillet of lower leg with fibula and tibia, free fillet of lower leg with fibula only, free fillet of lower leg with tibia only, free fillet of medial thigh, FVFG, other/unspecified), and outcomes.

### Outcomes and definitions

Flap survival was defined as absence of total flap loss; partial necrosis counted as a complication when specified but not as flap loss. Any complication was a composite of binary reporting (yes/no) as provided in source articles; specific complications (e.g., wound breakdown, infection) were captured qualitatively when counts were ambiguous.

### Synthesis methods

Given small, heterogeneous series and incomplete reporting, we estimated pooled proportions and Wilson 95% confidence intervals for overall and by‑category outcomes, restricted to records explicitly reporting the endpoint. Analyses were unadjusted and descriptive; we did not compute heterogeneity statistics or perform meta‑regression. As a sensitivity context, we report reporting completeness (fraction of cases/studies contributing to each endpoint) and avoid inferential comparisons between flap categories.

### Risk of bias and certainty

Most included reports were single‑center case reports or small series (Level IV). We therefore summarized reporting quality and completeness rather than applying comparative cohort tools. Certainty of evidence is very low due to study design and selective reporting.

## Results

### Study selection and yield

**Figure 9:**
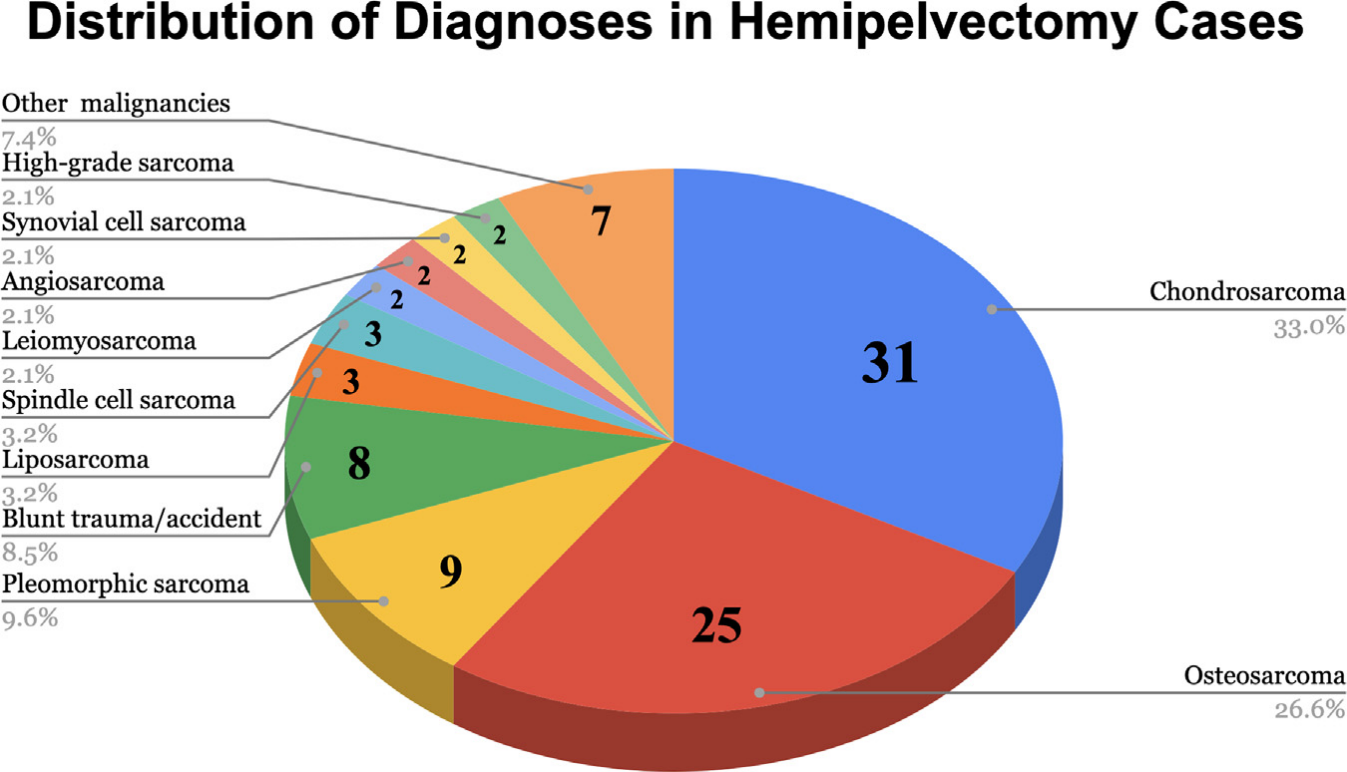
Distribution of diagnoses among reported hemipelvectomy cases. The most common indications were chondrosarcoma (33%) and osteosarcoma (27%), followed by pleomorphic sarcoma, trauma, and liposarcoma.

A total of 273 titled records were identified; after removing 26 duplicates, 247 unique records underwent screening, and 43 studies (94 cases) met inclusion criteria (Table 1). Exclusion reasons among 230 records included: no hemipelvectomy, no primary cases, pedicled-only reconstructions, non-English without extractable data, and unavailable full text (Figure 9).

**Table 1:** Summary of free fillet flap reconstructions after hemipelvectomy or transpelvic amputation. One row per article.

Citation	Cases	Sex (M/F)	Mean age	Side (L/R)	Flap type(s)	Diagnosis summary
Roulet et al.^4^	7	6/1	54	*L* = 4, *R* = 3	Sup. post. comp.(3), Leg+fibula(4)	OS(2), CS(3), PS(1), CCS(1)
Sawyer et al.^9^	1	0/1	50	*L* = 1	Leg+fibula(1)	PS(1)
Boehmler et al.^10^	1	1/0	55	*L* = 1	Leg+fibula+tibia(1)	Lipo(1)
Kreutz-Rodrigues et al.^2^	7	5/2	51	*L* = 2, *R* = 5	Fillet leg(6), Leg+fibula(1)	CS(4), PS(1), AS(1), Sarc NOS(1)
Bibbo et al.^11^	3	3/0	37	*L* = 1, *R* = 2	Fillet leg(3)	OS(1), CS(1), Leiomy(1)
Saksena et al.^12^	1	0/1	39	*L* = 1	Fillet leg(1)	Spindle(1)
Yamamoto et al.^13^	1	1/0	55	*R* = 1	Leg fillet(1)	CS(1)
Lauritzen et al.^14^	1	1/0	67	*L* = 1	Leg fillet(1)	PS(1)
Faisham et al.^15^	1	1/0	16	*R* = 1	Fillet leg(1)	Trauma(1)
Schiutema et al.^16^	1	1/0	21	*R* = 1	Leg fillet(1)	Trauma(1)
Faria et al.^17^	3	2/1	39	*L* = 2, *R* = 1	Fillet leg(3)	Lipo(1), FH(1), PS(1)
Saad et al.^18^	1	1/0	13	*L* = 1	Fillet leg(1)	OS(1)
Cigna et al.^19^	1	1/0	35	*L* = 1	Leg+fibula+tibia(1)	OS(1)
Reish et al.^20^	1	1/0	16	*R* = 1	Fillet tibia(1)	OS(1)
McKnight et al.^21^	1	0/1	22	*L* = 1	Leg+fibula(1)	OS(1)
Cavadas et al.^22^	1	1/0	18	*R* = 1	Fillet leg(1)	Trauma(1)
Kliski et al.^23^	11	7/4	52	–	Fillet leg(11)	OS(3), CS(3), PS(1), LS(1), LMS(1), others(2)
Kreutz-Rodrigues et al.^24^	7	3/4	41	*L* = 1, *R* = 6	Fillet leg(7)	CS(4), PS(1), AS(1), OS(1)
Yazawa et al.^25^	3	2/1	41	*L* = 2, *R* = 1	Medial thigh(2), Fillet flap(1)	OS(1), CS(1), Met(1)
Teven et al.^26^	1	1/0	36	*R* = 1	Fillet leg(1)	OS(1)
Miles et al.^27^	2	0/2	54	*R* = 2	Fillet leg(2)	PS(1), CS(1)
Movtchan et al.^28^	3	2/1	46	*R* = 3	Medial thigh(1), Leg fillet(1), Fillet leg(1)	OS(2), CS(1)
Young et al.^29^	1	1/0	58	*L* = 1	Leg fillet(1)	CS(1)
Anderson et al.^30^	2	2/0	45	–	Fillet leg(2)	CS(2)
Tashiro et al.^31^	6	–	65	–	Thigh fillet(3), Leg fillet(2), Fillet leg(2)	PS(3), CS(1), OS(1), SCC(1)
Ver Halen et al.^32^	7	4/3	35	–	Thigh fillet(3), Leg fillet(2), Fillet leg(2)	CRC(1), CS(3), Syn(1), SS(1), OS(1)
Newsome et al.^33^	1	1/0	20	*L* + *R*	Ant thigh(1)	OS(1)
Kamimoto et al.^34^	1	1/0	16	*R* = 1	Thigh fillet(1)	Trauma(1)
Cavadas et al.^35^	1	1/0	38	*L* = 1	Leg fillet(1)	OS(1)
Andreani et al.^36^	1	1/0	17	*L* = 1	Ant thigh(1)	OS(1)
Timmers et al.^37^	1	1/0	24	*L* = 1	Fillet leg(1)	OS(1), ES(1)
Burd et al.^38^	2	1/1	73	*L* = 2	Fillet leg(1)	CS(1)
Templeton et al.^39^	1	0/1	74	*R* = 1	Fillet leg(1)	CS(1)
Berner et al.^40^	1	1/0	36	*R* = 1	Leg fillet(1)	CS(1)
Bai et al.^41^	1	0/1	36	*R* = 1	Lat dorsi+ serratus(1), Lat dorsi(1)	Trauma(2)
Hung et al.^42^	2	1/1	65	*L* = 2	VFFG(1)	OS(1)
Pedreira et al.^43^	1	0/1	65	*L* = 1	Leg fillet(1)	CS(1)
Goulding et al.^44^	1	1/0	40	*R* = 1	Femur-fibula-fillet(1)	OS(1)
Rashid et al.^45^	1	1/0	45	*R* = 1	Fillet leg(1)	SS(1)
Kaiser et al.^46^	1	1/0	68	*R* = 1	Fillet leg(1)	OS(1)
Mueller et al.^47^	1	1/0	15	*R* = 1	Ant thigh(1)	OS(1)
Newsome et al.^48^	1	0/1	34	*R* = 1	Fillet leg+fibula(1)	OS(1)
Dvorak et al.^49^	1	0/1	34	*R* = 1	Fillet leg+fibula (1)	OS(1)

Abbreviations: OS, osteosarcoma; CS, chondrosarcoma; PS, pleomorphic sarcoma; CCS, clear cell sarcoma; LMS, leiomyosarcoma; AS, angiosarcoma; Lipo, liposarcoma; FH, fibrohistiocytoma; Syn, synovial sarcoma; SS, spindle sarcoma; SCC, squamous cell carcinoma; Met, metastasis; ES, Ewing sarcoma.

### Characteristics of the evidence base

The included publications spanned the years 1999 (inception) to 2025, with the median year of publication around 2017. Most reports were either single case descriptions or very small series, with a median of one case per study, an interquartile range of one to two, and a maximum of eleven cases in any individual report. At the case level, the median patient age was 45 years (interquartile range 30.8–61.3) among the 84 cases where age was documented. Sex was reported in 78 cases, of which 67% were male and 32% were female. Laterality was available in 65 cases, with the right side involved in 58%, the left side in 40%, and bilateral procedures in 1.5% (Table 2).

**Table 2:** Demographics of free-flap reconstructions after external hemipelvectomy (43 studies, 94 cases).

Characteristic	Findings
Publication years	1999–2025 (median ≈ 2017)
Cases per study	Median 1 (IQR 1–2); mean 2.19; max 11
Patient age	Study-level: median 39 (IQR 20–55); case-level: median 45 (IQR 30.8–61.3)
Sex distribution	Male 53/78 (67.9%); Female 25/78 (32.1%); completeness 83%
Laterality	Right 38/65 (58.5%); Left 26/65 (40.0%); Bilateral 1/65 (1.5%); completeness 69%

Study- and patient-level demographics of included reports of free flap reconstruction after external hemipelvectomy (*n* = 43 studies, 94 cases).

Abbreviations: IQR, interquartile range.

### Flap categories

**Figure 10:**
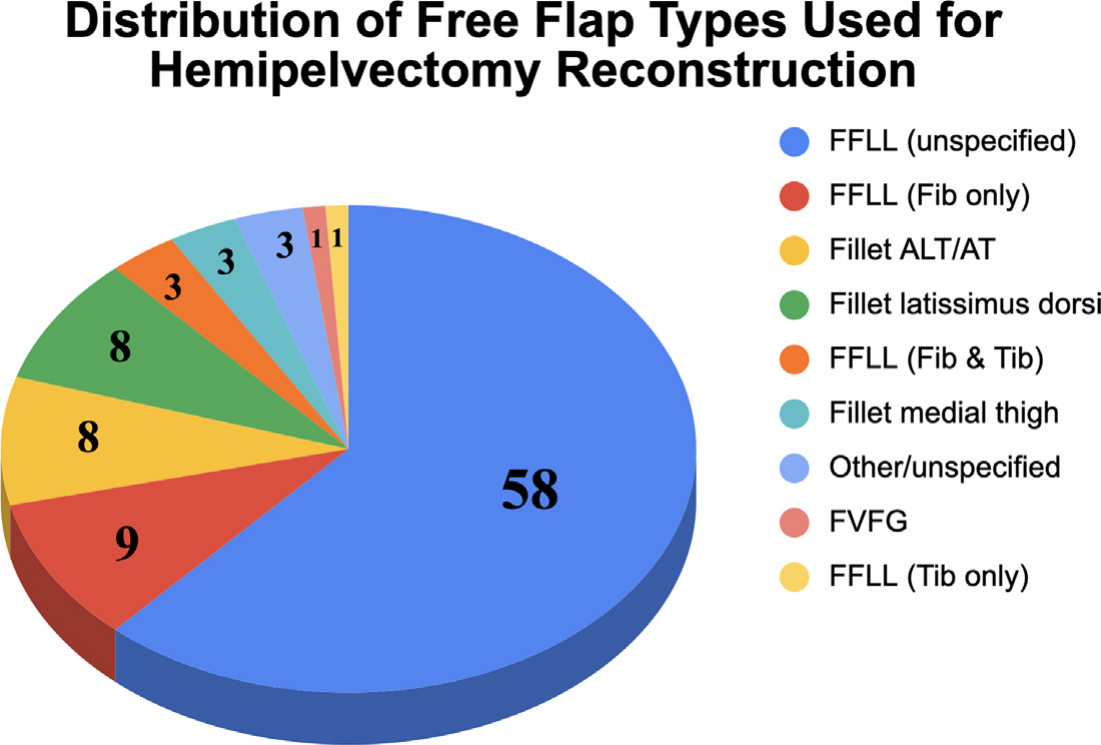
Distribution of free flap types used for hemipelvectomy reconstruction. Abbreviations: FFLL, free fillet of lower leg; Fib, fibula; Tib, tibia; ALT/AT, anterolateral/anterior thigh; FVFG, free vascularized fibular graft.

In terms of flap selection, the free fillet of lower leg was the most commonly used, representing 58 cases (62%). Additional reconstructions included fillet lower leg with fibula only in 9 cases (9.6%), anterolateral/anterior thigh (ALT/AT) in 8 cases (8.5%), latissimus dorsi in 8 cases (8.5%), and other/unspecified lower-extremity flaps in 3 cases (3.2%). Less frequent options were fillet lower leg with fibula and tibia (3 cases, 3.2%), fillet medial thigh (3 cases, 3.2%), FVFG alone (1 case, 1.1%), and fillet lower leg with tibia only (1 case, 1.1%) (Figure 10). The relative use of fillet-of-leg flaps increased steadily over time, rising from approximately 47% of reported cases in studies published up to 2014, to 57% between 2015 and 2020, and reaching 88% in reports published from 2021 to 2025 (Table 3).

**Table 3:** Flap distribution and era trends after external hemipelvectomy (*n* = 94).

Flap category	≤2014	2015–2020	2021–2025
Free fillet of lower leg (unspecified)	16	22	20
Free fillet of lower leg + fibula only	2	6	1
Free fillet of lower leg + fibula + tibia	1	1	1
Free fillet of lower leg + tibia only	1	0	0
Free fillet of medial thigh	2	0	1
Free fillet of ALT/AT	7	1	0
Free fillet of latissimus dorsi	2	6	0
FVFG	0	1	0
Other/unspecified	0	3	0
Total cases per era	31	40	23

Distribution of flap categories and temporal trends among 94 reported free flap reconstructions after external hemipelvectomy, stratified by publication era.

Abbreviations: ALT/AT, anterolateral/anterior thigh; FVFG, free vascularized fibular graft.

### Overall outcomes

Among cases where flap survival was explicitly reported, 65 of 69 flaps were successful, yielding a pooled survival rate of 94% (95% CI 86.0–97.7). For postoperative complications, 28 of 64 reported cases experienced at least one adverse event, corresponding to a pooled complication rate of 44% (95% CI 32.3–55.9). Reporting completeness was 73% for survival (69/94 cases) and 68% for complications (64/94 cases) (Table 4, Table 5, Table 6).

**Table 4:** Overall flap survival and complications.

Outcome	Numerator/Denominator	Rate (%)	95% CI
Flap survival	65/69	94.2	86.0–97.7
Any complication	28/64	43.8	32.3–55.9
Reporting completeness (studies)	31/43 (72.1%)		Survival endpoint
Reporting completeness (cases)	69/94 (73.4%)		Survival endpoint
Reporting completeness (studies)	32/43 (74.4%)		Complication endpoint
Reporting completeness (cases)	64/94 (68.1%)		Complication endpoint

Overall flap survival and complication rates among reported cases, including numerators, percentages, and 95% confidence intervals (CIs), with reporting completeness for survival and complication endpoints.

**Table 5:** Flap survival after external hemipelvectomy (n/N, %, 95% CI).

Flap category	Survival (n/N)	Rate (%)	95% CI
Free fillet of lower leg (unspecified)	36/38	94.7	82.7–98.5
Fillet lower leg w/fibula only	7/8	87.5	52.9–97.8
Fillet lower leg w/fibula + tibia	3/3	100.0	43.9–100.0
Fillet medial thigh	2/3	66.7	20.8–93.9
Fillet ALT/AT	7/7	100.0	64.6–100.0
Fillet latissimus dorsi	6/6	100.0	61.0–100.0
Vascularized fibular graft (FVFG)	1/1	100.0	20.7–100.0
Fillet lower leg w/tibia only	—	DNS	—
Other/unspecified	3/3	100.0	43.9–100.0

Survival rates by flap type among reported free flap reconstructions after external hemipelvectomy.

Abbreviations: ALT/AT, anterolateral/anterior thigh; FVFG, free vascularized fibular graft; DNS, data not specified; CI, confidence interval.

**Table 6:** Complication rates by flap type after hemipelvectomy.

Flap type	Complications (n/N)	Rate (%)	Phenotypes (reported)
Free fillet of lower leg (unspecified)	14/34	41.2	Wound dehiscence, infection
Fillet lower leg w/fibula only	5/7	57.1	Wound infection, flap loss
Fillet lower leg w/fibula + tibia	2/3	66.7	Wound breakdown, venous stasis
Fillet medial thigh	1/3	33.3	Venous thrombosis, transfusion protocol
Fillet ALT/AT	1/7	14.3	Retroperitoneal abscesses
Fillet latissimus dorsi	3/6	50.0	Abscess formation
Vascularized fibular graft (FVFG)	1/1	100.0	Wound infection
Fillet lower leg w/tibia only	–	DNS	–
Other/unspecified	3/3	100.0	Infection, necrosis

Complication rates by flap type among reported free flap reconstructions after external hemipelvectomy.

Abbreviations: ALT/AT, anterolateral/anterior thigh; FVFG, free vascularized fibular graft; DNS, data not specified.

### Outcomes by flap type

When broken down by flap type, survival was high across most categories. Free fillet of lower leg (unspecified) achieved 36/38 survivals (95%); ALT/AT and latissimus dorsi both had 100% survival (7/7 and 6/6, respectively). Fillet lower leg with fibula and tibia and the other/unspecified category each had 100% survival (3/3), as did the single FVFG case (1/1). In contrast, fillet lower leg with fibula only had 7/8 survivals, and the fillet medial thigh subgroup had 2/3 survivals. The single tibia-only fillet case did not have survival data reported.

### Complications by flap type

Complication rates varied by technique. Free fillet of lower leg (unspecified) had 14/34 cases with complications; ALT/AT was 1/7; latissimus dorsi was 3/6; fillet lower leg with fibula only was 5/7; fillet lower leg with fibula and tibia was 2/3; and fillet medial thigh was 1/3. Both the FVFG (1/1) and other/unspecified groups (3/3) reported complications in all cases with available data, while the tibia-only fillet did not report complications. Across studies that detailed individual adverse events, wound breakdown or dehiscence and local infection were the most frequently encountered complications, while partial skin necrosis and abscess formation appeared less consistently. Rare systemic complications such as sepsis, myocardial infarction, and thromboembolic events (including DVT or pulmonary embolism–related collapse) were also recorded.

### Reporting completeness and sensitivity context

Approximately 73% of cases contributed to survival estimates and 68% to complication analyses; even fewer provided both within the same cohort. Given small denominators in several subgroups and variable reporting completeness, between-category differences should be interpreted cautiously and considered hypothesis-generating.

## Discussion

This combined pediatric case and systematic review supports the free fillet of leg flap as a reliable, donor-site–sparing solution for hemipelvectomy defects, a category of reconstruction that remains among the most technically demanding in modern oncologic surgery. The analysis of 43 studies encompassing 94 cases showed that overall survival of free flaps in this setting exceeded 95% among cases with explicit reporting, and survival for fillet flaps was uniformly high across the reported cohorts. This level of consistency is striking given the heterogeneity of the underlying population, the oncologic indications, and the wide temporal span of the literature. The high survival rate strongly suggests that when the procedure is technically feasible, the FFLF provides a level of reliability that is not easily matched by alternative free-flap choices. At the same time, overall complications approached 50%, with wound-related events most frequently described. These findings underscore that the challenge lies not in flap perfusion alone but in the broader context of hemipelvectomy as a radical operation that disrupts anatomy, physiology, and postoperative function.

The pediatric case presented in this report highlights the practical relevance of these findings and illustrates how the principles identified in the systematic review can be applied at the individual level. In our patient, an 11-year-old boy with recurrent inflammatory myofibroblastic tumor, the FFLF offered a unique advantage by obviating the need for a secondary donor site and providing sufficient bulk to protect exposed viscera and facilitate early sitting and ambulation. This donor-site–sparing characteristic is especially valuable in children, where long-term morbidity of alternative donor sites may have decades of consequences, and where body size can limit the options for other large-volume free flaps.^50^ The use of simultaneous harvest during extirpation minimized ischemia time, the preparation of recipient vessels in the iliac system ensured unobstructed inflow and outflow, and mesh reinforcement of the abdominal wall created a stable platform for inset. When postoperative venous congestion developed due to ileus and intra-abdominal pressure, prompt recognition, aggressive bowel decompression, and the adjunct of medicinal leech therapy salvaged the flap. These strategies, all supported by principles echoed in the review, illustrate how technical execution and perioperative vigilance remain paramount even when a flap with excellent survival characteristics is chosen.

From a practical standpoint, the FFLF should be considered in scenarios where local pedicled options are unavailable due to oncologic resection, prior irradiation, or inadequate bulk. The flap is particularly advantageous when large volumes of tissue are required to obliterate pelvic dead space or to protect critical abdominal and retroperitoneal structures.^24^^,^^51^ Minimizing donor-site morbidity is another compelling reason to select this approach, especially in pediatric or otherwise frail patients.^52^^,^^53^ However, the extent of tumor involvement and the margin requirements may restrict the availability of suitable fillet flap tissue or limit pedicle length, making this an important preoperative factor when comparing alternative reconstructive options. FFLF should be avoided when the amputated limb or its vascular pedicle is involved by tumor or required oncologic margins, or when the limb tissue is nonviable/contaminated such that reliable perfusion cannot be ensured.

In our synthesis, fillet flaps not only demonstrated excellent survival but also avoided the creation of a second major wound, which can reduce pain, shorten hospitalization, and simplify rehabilitation. To execute the procedure effectively, careful coordination between surgical teams is essential.^7^ Synchronizing flap harvest with tumor resection reduces ischemia; extended pedicle dissection facilitates tension-free microvascular anastomosis to suitable recipient vessels; acellular or synthetic mesh placement provides a barrier protecting pelvic viscera; and measures to prevent venous congestion enhance flap viability.^26^ Although leech therapy is not without its drawbacks, including transfusion requirements and infection risks, it remains a pragmatic salvage adjunct in the event of postoperative venous congestion.

Children pose unique challenges for hemipelvectomy reconstruction, and our case adds valuable insight to the very limited literature in this population. Pediatric patients have smaller-caliber vessels, lower blood volumes, and a higher susceptibility to hemodynamic swings during long procedures.^54^^,^^55^ Rehabilitation needs are also distinct, requiring careful coordination with prosthetists, physical therapists, and psychosocial support teams.^56^ Our patient demonstrated that with early planning and a structured rehabilitation pathway, rapid wound healing and prosthetic-assisted ambulation are achievable. At 6 weeks, the wound was fully epithelialized, and at 5 months he was ambulating with a prosthesis and one crutch, an outcome that speaks to both the functional success of the reconstruction and the resilience of pediatric patients when supported by a multidisciplinary team. The case therefore expands the age spectrum for which FFLF can be considered and reinforces its applicability in younger populations where donor-site preservation is particularly critical.

Because this reconstruction was performed in a growing child, long-term outcomes must be considered in the context of ongoing skeletal development. Although the free fillet-of-leg flap primarily provides soft-tissue coverage rather than restoring bony pelvic growth, children may experience evolving pelvic obliquity, compensatory spinal alignment changes, and shifting sitting/prosthetic interface demands as they mature. Accordingly, we emphasize surveillance through skeletal maturity with pediatric orthopedics and rehabilitation, with anticipatory prosthetic refitting and ongoing monitoring of skin integrity and functional alignment.

When the FFLF is compared to other free flaps reported in the literature, the advantages become clearer. Flap selection follows a simple extent-and-location algorithm: when imaging confirms distal tissues and the popliteal pedicle are uninvolved, FFLF is preferred for large central/posterior defects needing bulk, whereas proximal tumor extension or primarily anterior/cutaneous needs favor conventional free flaps (e.g., ALT or latissimus), linking resection planes directly to flap choice for preoperative planning.

Alternative options such as the latissimus dorsi, anterolateral thigh, or medial thigh flap can yield acceptable survival in selected patients but introduce additional donor-site morbidity.^4^ An ALT flap may be preferable when a thinner, more pliable soft-tissue envelope is needed for perineal contouring and hygiene access, or when spared-part tissue cannot be used due to limb involvement within the resection field. A latissimus dorsi flap may be favored when broad, reliable well-vascularized muscle coverage is required and the amputated limb is unavailable or unsuitable for fillet reconstruction.^4^ The anterolateral thigh flap can provide skin and soft tissue but may not deliver the volume necessary to obliterate large pelvic cavities.^57^ Medial thigh flaps often lack the reach and bulk required for deep pelvic or perineal reconstruction, limiting their utility in extensive defects. The FFLF, in contrast, provides generous tissue with consistent vascular anatomy and no new donor wound.^5^ However, the flap’s bulk and early volume changes can complicate socket fabrication and increase shear risk, necessitating early prosthetist input, staged sockets, and delayed definitive fitting until the flap stabilizes. In an era where enhanced recovery and patient-centered outcomes are increasingly emphasized, these characteristics give the FFLF a clear edge.

Moreover, integration with modern reconstructive techniques such as targeted muscle reinnervation or regenerative nerve interfaces could further improve functional outcomes when coaptations are possible in the residual trunk.^5^ These strategies, while not widely reported in the current literature, represent promising adjuncts for improving comfort, reducing neuroma formation, and optimizing prosthetic interface.

Despite the positive findings, the evidence base is not without limitations. The majority of included reports were case reports or small case series, which inherently limits generalizability and increases the risk of publication bias. Reporting was often incomplete, with 73% of cases providing survival data (69/94) and 68% providing complication data (64/94). This selective reporting creates uncertainty and prevents robust comparisons between flap categories. We addressed this by restricting pooled estimates to explicitly reported endpoints and by presenting Wilson confidence intervals along with reporting completeness metrics, thereby contextualizing the uncertainty rather than overstating the precision of the findings. Nevertheless, the conclusions should be considered hypothesis-generating rather than definitive. Larger, prospective, multicenter registries would be valuable in establishing standardized outcome definitions.

Future research should also explore perioperative management protocols tailored to the unique challenges of hemipelvectomy patients. In particular, strategies for intra-abdominal pressure management may play a critical role in reducing postoperative venous congestion, as demonstrated in our pediatric case. Enhanced recovery protocols that integrate early mobilization, aggressive bowel regimens, and standardized monitoring could help mitigate the high complication rates observed in the literature. Furthermore, long-term outcomes such as prosthetic use, return to work or school, and psychosocial adjustment remain underreported yet are highly relevant to patients and families.

Another important consideration is the temporal trend observed in our review, with the proportion of fillet-of-leg flaps increasing steadily over time and reaching 67% of cases reported within the last decade (2015–2025). This suggests growing confidence among surgeons in the reliability and utility of the FFLF for hemipelvectomy coverage. The trend also reflects a broader movement in reconstructive surgery toward donor-site–sparing approaches that maximize function and minimize morbidity.

## Conclusion

In conclusion, the free fillet of leg flap provides robust and reliable coverage for external hemipelvectomy defects, with high reported survival rates and the significant advantage of avoiding a secondary donor site. The pediatric case presented here illustrates its feasibility and functional success in a younger population, where donor-site preservation and rapid rehabilitation are particularly valuable. Although complications remain common, they are largely attributable to the magnitude of the underlying procedure rather than flap-specific failure. With meticulous surgical planning, intraoperative coordination, and vigilant postoperative care, many of these risks can be mitigated. The systematic review underscores the need for standardized reporting and prospective data collection to strengthen the evidence base. Nevertheless, current data and clinical experience suggest that the FFLF should be considered a leading option for hemipelvectomy reconstruction, with particular promise for pediatric and high-risk populations. By continuing to refine technical strategies and enhance perioperative care, reconstructive surgeons can further improve outcomes and quality of life for this challenging patient group.

## Funding

This research received no external funding.

## Ethical approval

Not required.

## Declaration of competing interest

The authors declare no conflicts of interest.
